# NETosis: an emerging therapeutic target in renal diseases

**DOI:** 10.3389/fimmu.2023.1253667

**Published:** 2023-09-08

**Authors:** Márk Juha, Adél Molnár, Zoltán Jakus, Nóra Ledó

**Affiliations:** ^1^ Department of Internal Medicine and Oncology, Semmelweis University, Budapest, Hungary; ^2^ Department of Physiology, Semmelweis University, Budapest, Hungary

**Keywords:** NETosis, neutrophil extracellular traps, renal diseases, lupus nephritis, ANCA associated vasculitis

## Abstract

**Introduction:**

Neutrophil extracellular traps (NETs) are web-like structures composed of nuclear and granular components. The primary role of NETS is to prevent the dissemination of microbes and facilitate their elimination. However, this process is accompanied by collateral proinflammatory adverse effects when the NET release becomes uncontrollable, or clearance is impaired. Although NET-induced organ damage is conducted primarily and indirectly via immune complexes and the subsequent release of cytokines, their direct effects on cells are also remarkable. NETosis plays a critical pathogenic role in several renal disorders, such as the early phase of acute tubular necrosis, anti-neutrophil cytoplasmic antibody-mediated renal vasculitis, lupus nephritis, thrombotic microangiopathies, anti-glomerular basement membrane disease, and diabetic nephropathy. Their substantial contribution in the course of these disorders makes them a desirable target in the therapeutic armamentarium. This article gives an in-depth review of the heterogeneous pathogenesis and physiological regulations of NETosis and its pivotal role in renal diseases. Based on the pathogenesis, the article also outlines the current therapeutic options and possible molecular targets in the treatment of NET-related renal disorders.

**Methods:**

We carried out thorough literature research published in PubMed and Google Scholar, including a comprehensive review and analysis of the classification, pathomechanisms, and a broad spectrum of NET-related kidney disorders.

**Conclusions:**

NETosis plays a pivotal role in certain renal diseases. It initiates and maintains inflammatory and autoimmune disorders, thus making it a desirable target for improving patient and renal outcomes. Better understanding and clinical translation of the pathogenesis are crucial aspects to treatment, for improving patient, and renal outcomes.

## 1 Introduction

Neutrophil granulocytes are crucial members of innate immunity. Their antimicrobial arsenal includes 1) the release of granular proteins such as neutrophil elastase (NE) and myeloperoxidase (MPO), 2) the phagocytosis via the production of reactive oxygen species (ROS) inside the phagosome, and 3) the formation of neutrophil extracellular traps (NETs) (1–3).

NETs are web-like structures of nuclear and granular components released from the membrane of activated neutrophils. The granular components are the contents of the primary (azurophilic) granules, such as NE, cathepsin G, MPO, and LL-37 (cathelicidin) as well as the secondary and tertiary granules (lactoferrin, gelatinase) (4). Highly decondensed chromatin fibers and citrullinated histone proteins make up the majority of the nuclear components. Four main forms of NETosis (the mechanism of releasing NETs) are known today: lytic (suicidal), non-lytic (vital), caspase 11/4-mediated, and mitochondrial NETosis.

Traditionally, cell death is categorized either as accidental or programmed cell death. Accidental cell death – *necrosis* – is an uncontrolled process that occurs as a result of an overwhelming stimulus that is accompanied by inflammatory responses caused by releasing components of the dying cell such as heat shock proteins, uric acid, and nuclear proteins. Programmed cell deaths include apoptosis, pyroptosis, necroptosis, and autophagy. *Apoptosis* occurs in non-inflammatory conditions and is precisely conducted by a cascade of molecular events (membrane blebbing, size reduction, chromatin condensation, and DNA fragmentation) ended with the engulfment of the granule-packed cellular components by phagocytes, without breaking the cell membranes (5). *Pyroptosis* is a process, driven by the inflammasome and Gasdermin-D, during which the cell swells until its membrane eventually breaks down (6). *Necroptosis* is a lytic form of cell death and mimics both the characteristics of apoptosis and necrosis and it is mediated by the receptor-interacting protein kinase (RIPK3) and the mixed lineage kinase domain-like (MLKL) pseudokinase. During *autophagy*, the primary role is to meet metabolic needs and to recycle certain cytoplasmic proteins and organelles by engulfing and covering them in vesicles that fuse with lysosomes to digest their contents. In contrast to the other canonical forms of programmed cell death, NETosis can proceed differently depending on the stimuli and the environment (7, 8).

The first evidence of NETosis was described in 1996 by Takei et al., who found that phorbol-12-myristate-13-acetate (PMA)-induced neutrophils died by a previously unknown mechanism other than apoptosis or necrosis (9). Later, in 2004, Brinkmann et al. revealed that activated neutrophils release their nuclear and granular contents to the extracellular space, where they form a web-like network to immobilize and kill bacteria (4). It is well-known that NETs can bind microbes to prevent them from spreading and eliminate them, but they may also convey unfavorable effects in tissue damage, atherosclerosis, thrombosis, or malignancies (10–12).

Furthermore, the role of NETosis was already described in autoimmune conditions. Enhanced production or decreased clearance of the NETs causes dysregulated immunity, autoantigen modification and externalization, and tissue damage (13). The importance of NETosis has been demonstrated in the pathogenesis of a variety of disorders, such as systemic lupus erythematosus (SLE), rheumatoid arthritis (RA), antineutrophil cytoplasmic antibody (ANCA)-associated vasculitis (AAV), psoriasis and gout (14).

In this review article, we summarize the heterogeneous pathogenesis and physiological regulations of NETosis and its pivotal role in renal diseases. Based on the pathogenesis, the article also discusses current therapeutic options and possible molecular targets in the treatment of NET-related renal disorders.

## 2 Pathomechanisms

### 2.1 Lytic (suicidal) NET formation

**Figure 1:**
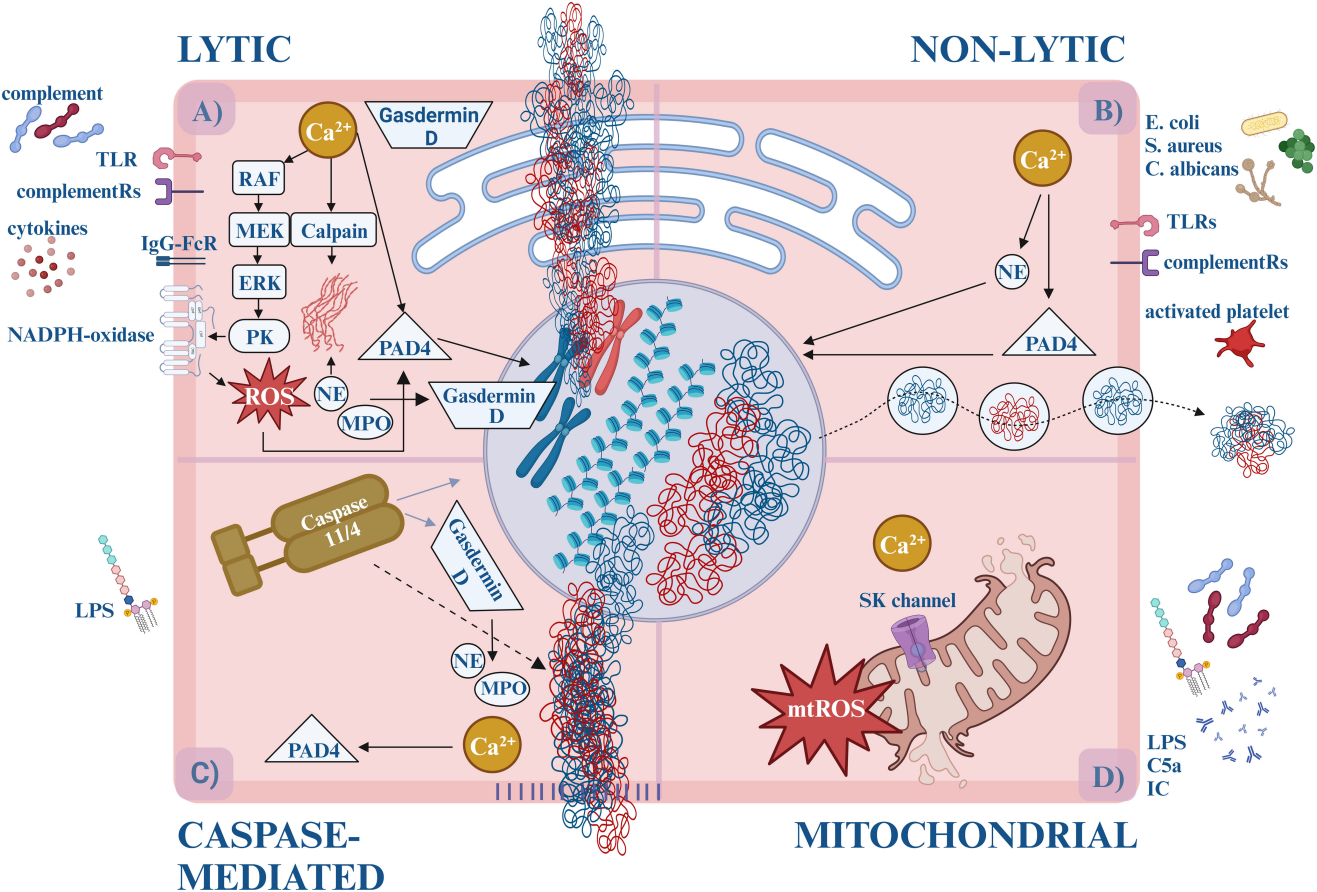
The forms of NETosis. **(A)** Lytic (suicidal) NET formation. By the activation of TLR (e.g., TLR2, TLR4, TLR7, TLR9) or IgG-Fc receptors (FcγRIIa and/or FcγRIIIb) calcium is released from the ER, activating PKC via the RAF-MEK-MAPK/ERK pathway. PKC triggers the assembly of the membrane-bound subunits of NADPH-oxidase, which starts generating ROS. ROS and/or calcium activates PAD4, which translocates to the nucleus and induces the citrullination of arginine amino acids, loosening the histone-DNA interaction, and inhibits the H1 histone-mediated compaction causing chromatin decondensation. Simultaneously, enzymes (MPO, NE) are released from the granules. NE and the calcium-activated calpain degrade the cytosolic filamentous network and the nuclear lamina. With the participation of Gasdermin-D, NE destructs the nuclear envelope and enables water influx. NE cleaves histones causing further chromatin decondensation. Eventually, Gasdermin D-mediated pore opening of the plasma membrane leads to the externalization of the DNA, cytosolic and granular proteins. **(B)** Non-lytic (vital) NET formation. Different stimuli induce non-lytic NET formation, like bacteria, and fungi via TLRs (e.g., TLR2, TLR4, TLR7, TLR9) or complement receptors (e.g., CR1, CR3, CR4, C5aR1). Calcium influx starts through the SK-channels leading to PAD4 and NE activation. They translocate to the nucleus and decondense the chromatin. Unlike lytic NETosis, NADPH oxidase is not involved in the process, and the nuclear content is released from the cell in vesicles. It takes approximately 5-60 minutes, compared to the lytic form, which takes about 3-4 hours. **(C)** Caspase-mediated NETosis. LPS activates caspase-11/4 via an uncanonical inflammasome pathway. Then caspase-11/4 activates Gasdermin D, which starts forming pores on the nuclear-, plasma- and granular membranes, enabling calcium influx and NE, MPO release. Caspase-11/4 enters the nucleus and cleaves histones, while the calcium-activated PAD4 citrullinates the arginine amino acids. Although NE, MPO, and PAD4 facilitate the process, their activities are not prerequisites for NETosis. **(D)** Mitochondrial NETosis. LPS, GM-CSF, C5a or IC induction elevates cytosolic calcium level, which opens SK channels and leads to mtROS production, and mitochondrial DNA release. ER, endoplasmatic reticule; DNA, deoxyribonucleic acid; GM-CSF, granulocyte-macrophage colony-stimulating factor; IC, immune complexes; IgG-FcR, immune globulin G – fragment crystallizable receptor; LPS, lipopolysaccharides; MPO, myeloperoxidase; mtROS, mitochondrial reactive oxygen species; NAPDH oxidase, nicotinamide adenine dinucleotide phosphate oxidase; NE, neutrophil elastase; PAD4, protein arginine deiminase 4; PKC, protein kinase-C; ROS, reactive oxygen species; SK-channels, small conductance calcium-activated potassium channels; TLR, Toll-like receptor.

The most extensively studied type of NET formation is called “suicidal” or “lytic”, also referred as *NETosis*. This is indeed a form of cell death because once the neutrophils release their contents, they eventually perish (**Figure 1A**).

Based on the involvement of nicotinamide adenine dinucleotide phosphate (NADPH)-oxidase, lytic NETosis was used to be divided into *NADPH-dependent* and *NADPH-independent* forms. Later it became clear that the NADPH-independent pathway is conducted by mitochondrial ROS (mtROS) production, hence, the term, “NADPH-independent” was modified to “mitochondria-dependent NETosis” (15). On the other hand, since NADPH-oxidase may be induced by mtROS - NADPH-oxidase can engage in both processes.

Furthermore, triggering stimuli have a pivotal role and the signalization of NETosis depends on them. Proteomic analysis showed diversity in terms of NET-constitution and post-translational modifications, which suggests that the stimulus determines the biological effects (16).

Conventional lytic NETosis is usually initiated by complement proteins (such as C3b, C5a), cytokines, and ligands binding to Toll-like receptors (TLR) or IgG-Fc receptors (17). Upon receptor activation, calcium storage is released from the endoplasmic reticulum. Increased cytoplasmic calcium levels activate protein kinase C (PKC) via the RAF-MEK-MAPK/ERK signaling pathways and induce the phosphorylation of gp91phox. This stimulates the membrane-bound subunits of NADPH-oxidase to assemble into the functional enzyme in the cytoplasmic or phagosome membranes. Thus, the reactive oxygen species (ROS) generation is initiated (18, 19).

Simultaneously, the disintegrated azurophilic granules, MPO, NE, and other lytic enzymes are released into the cytosol. By oxidizing it, MPO facilitates the release of NE into the cytoplasm, however, the exact role of the lytic enzymes in this process still needs to be elucidated (20, 21).

The cytosolic fibrous network also falls victim to degradation. The disassembly of actin and vimentin molecules is promoted by calcium influx. Activated by calcium level increase and citrullination, calpain - a serine protease – facilitates the decondensation of micro- and intermedier filaments and nuclear lamin as well (22). The intactness of the microtubular network, however, does not affect the NETosis (23). NE binds and degrades the actin fibers (preventing concurrent phagocytosis), thus making its way to the nucleus. Cooperating with Gasdermin-D, NE destructs the nucleus envelope and enables water influx (21). Here NE proceeds to cleave histones which eventually leads to chromatin decondensation (24–26).

By binding calcium ions or activated by ROS, protein-arginine deiminase 4 (PAD4) adopts its catalytically active form. PAD4 translocates to the nucleus which induces citrullination of the arginine amino acids, thus reducing the positive charge and loosening the histone-DNA interaction. PAD4 also has direct effects on chromatin decondensation via inhibition of linker histone-mediated compaction (23). Ultimately, these processes lead to the decondensation of the chromatin structure (27, 28). Gasdermin D-mediated pore openings induce the progressive permeabilization of the plasma membrane which leads to the externalization of the DNA, cytosolic and granular proteins (29). Of note, pore formation changes the intracellular calcium gradient, which further promotes PAD4 activation (30).

### 2.2 Non-lytic (vital) NET formation

During “vital” or “non-lytic” NET formation neutrophils maintain their viability and antimicrobial functions (e.g., engaging in recruitment, chemotaxis, and phagocytosis) after they release the nuclear or mitochondrial DNA (**Figure 1B**).

Non-lytic NETosis is initiated by the detection of stimuli of complement receptors and activated platelets. Staphylococcus aureus, Escherichia coli, and Candida albicans can also cause non-lytic NET formation by activation of complement receptor (CR) -1, -3, -4 and TLR - 2, -4 ligands NADPH independently. Calcium influx starts through the small conductance potassium channel member three (SK) and activates PAD4 and NE. They translocate to the nucleus where they decondense the chromatin. Eventually, chromatin is expelled to the extracellular space by vesicular transport, in which the blebs fuse with the plasma membrane again. This mechanism maintains the integrity of the plasma membrane and the anucleated neutrophils (cytoplasts) stay alive, keeping their ability to do their antimicrobial functions: migrate and phagocytose. This process takes approximately 5-60 minutes, compared to the lytic form of NETosis, which requires 3-4 hours (20).

### 2.3 Other types of NETosis

The third form of NET formation is conducted by caspases. Cytosolic lipopolysaccharide (LPS) induction of neutrophils activates murine caspase-11 or the human ortholog caspase-4 via an uncanonical inflammasome pathway to enable Gasdermin D cleavage into the pore-forming fragments. Upon entering the nucleus, caspase-11/4 degrades the histones and chromatin. Gasdermin D forms pores on granules as well, liberating NE/MPO. Calcium influx through the pores activates PAD4 and citrullinates histones. Although NE, MPO, and PAD4 facilitate the process, their activities are not prerequisites for NETosis – caspase-induced chromatin cleavage converges in a similar molecular pathway as lytic NETosis (31) (**Figure 1C**).

Another pathway that leads to NETosis is the Ca^2+^-ionophore-induced mitochondrial ROS (mtROS) production. Elevated cytosolic calcium level opens SK channels, mediates mtROS production, and induces apoptosis and NET formation (15, 32). Granulocyte-macrophage colony-stimulating factor (GM-CSF), LPS, complement component 5a (C5a), or ribonucleoprotein-containing immune complexes also activate neutrophils to release mitochondrial DNA and ROS (33). Oxidized mtDNA has potent proinflammatory and type I interferon stimulatory properties that seem to play a pivotal role in the pathogenesis of lupus nephritis (LN) (34). Although mtROS-induced NETosis is known as NADPH-oxidase-independent NETosis, the crosstalk with NADPH-oxidase to produce additional ROS has already been demonstrated (32) (**Figure 1D**).

In conclusion, the contribution of PAD4 and ROS are the most relevant factors to group the processes. Lytic NETosis is ROS-dependent, and the participation of PAD4 is not obligatory, while non-lytic NET formation occurs in the absence of ROS with the important role of PAD4. **Table 1** summarizes the main characteristics of NET formation.

**Table 1:** Comparison of NETosis forms.

	Lytic (suicidal)	Non-lytic (vital)	Caspase-mediated	Mitochondrial
**Duration**	3-4 hours	50-60 minutes	depends on stimuli	depends on stimuli (C. albicans: 30 min; mito-antigens: 3-4h)
**Stimuli**	crystals (cholesterol, monosodium urate, calcium carbonate) auto-Abs, ICs viruses, bacteria, fungi, parasites DAMPS (histone, LDL) protein fibers tumor cells cytokines (TNFa, CXCL2, IL-1, IL-8, IL-18) LPS, activated platelets, PAF NO, H2O2 PMA	Bacteria: E. coli, S. aureus Fungi: C. albicans LPS IL-8 PAF ICs complements (e.g., C3b, C5a)	cytosolic LPS cytosolic G(-) bacteria	Ca^2+^-ionophores GM-CSF LPS C5a ribonucleoprotein-containing ICs UV
**Receptors**	TLRs (e.g., TLR2, TLR4, TLR7, TLR9), NOD-like Rs C-type lectin Rs, complement Rs, FcRs (e.g., FcγRIIa and/or FcγRIIIb), chemokine Rs (e.g., CXCR1, CXCR2 and CXCR4) Siglec, RAGE, PSGL1	CRs (e.g., CR1, CR3, CR4, C5aR1) TLRs (e.g., TLR2, TLR4, TLR7, TLR9) activated platelets	to be identified	to be identified TLRs (e.g., TLR7, TLR9) CRs (e.g., C5aR1)
**ROS**	dependent	independent	independent (see details in text)	mtROS-dependent
**PAD4**	independent	dependent	not obligatory	
**NE**	dependent		not obligatory	
**MPO**	dependent		not obligatory	
**In disease**	AAV		sepsis, RA, cancer	SLE, LN

Empty cells indicate that no matching data was found in the literature. AAV, anti-neutrophil cytoplasmic antibody (ANCA)-associated vasculitis; C5a, complement 5a; CR, complement receptor; CXCL2, chemokine (C-X-C motif) ligand 2; CXCR, chemokine receptor; DAMP, damage-associated molecular patterns; GM-CSF, granulocyte-macrophage colony-stimulating factor; H2O2, hydrogen peroxide; ICs, immune complexes; IL, interleukin; mtROS, mithocondrial reactive oxygen species; LN, lupus nephritis; LPS, lipopolysaccharides; NE, neutrophil elastase; NO, nitrogen oxide; NOD, nucleotide oligomerization domain; PAD4, protein arginine deiminase 4; PAF, platelet-activating factor; PMA, phorbol-12-myristate-13-acetate; PSGL-1, P-selectin glycoprotein ligand-1; R, receptor; RA, rheumatoid arthritis; RAGE, receptor for advanced glycation end products; ROS, reactive oxygen species; SLE, systemic lupus erythematosus; TLR, Toll-like receptor; TNF-α, tumor necrosis factor alfa; UV, ultraviolet.

### 2.4 Clearance of NETs

The control of NET formation and elimination is inevitable to maintain tissue homeostasis. Lytic neutrophil remnants are mainly taken up by macrophages. NET-contents function as danger-associated molecules and they are recognized by membrane-bound receptors (intracellular adhesion molecule 1, 3, liver X receptor, Mer tyrosine kinase) or soluble pattern recognition proteins (galectin-3, C3, C1q, annexin A1, factor-H related protein, C-reactive protein, clusterin, milk fat globule EGF factor 8). Scavenging macrophages identify NETotic neutrophils by “eat-me” signals or by the loss of “don’t eat-me” signals and phagocytosis takes place. Interestingly, neutrophil LL-37 interacts with extracellular anionic molecules such as dsDNA or dsRNA and aids their uptake by macrophages. Typically, the degradation of the apoptotic (and the NETotic) neutrophils creates a net anti-inflammatory microenvironment, hence this process differs from the traditional phagocytosis, so the term, “efferocytosis” was coined (35). After NETosis, interferons and other proinflammatory cytokines prime macrophages to M1 macrophages. M1 macrophages are characterized by the ability to secrete inflammatory cytokines and costimulatory molecules. Furthermore, upon interaction with NETs, M1 macrophages release DNA in a PAD4-dependent manner (36). Hence, they not only phagocyte the intercellular debris but also exacerbate inflammation. However, over time, M1 macrophages also activate their own caspase-activated DNase to degrade the surrounding extracellular DNA, and, the macrophage phenotype shifts toward an anti-inflammatory effector function, to the M2 macrophages (36). Although the molecular background of phenotype shifting is not fully unraveled, a crosstalk between the M1 and M2 polarizing pathways is proved. The increase of cell death by M1 macrophages is recognized by M2 phenotypes and they establish a predominantly anti-inflammatory milieu, therefore, the microenvironment commits to tissue remodeling and immune tolerance (37–39). Interestingly, M2 macrophages also contribute to the initial M1 polarization by releasing inflammatory cytokines upon encountering NETs (36). This highlights that macrophages could be further subdivided based on their functionally distinct roles (40).

The complement system also facilitates the clearance of cellular debris. All three complement pathways are involved in the removal of NETs; however, the classical pathway components, C3, C4, C5, and C1q have a higher affinity toward secondary necrotic cells like NETs. They either interact directly with DNA and mitochondrial DAMPs (damage-associated molecular patterns) or via immune complexes. Complement members do not only take part in opsonization but also aid the removal of NET remnants by activating serine proteases C1r and C1s (35).

Since DNA forms the backbone of NETs, DNases are central participants in degradation and digestion. The DNase complex contains three different enzymes (DNase I, DNase II, and DNase1-like 3 protein) with different functions (41). DNase I is responsible for the removal of protein-free DNA; while DNase1-like 3 protein degrades protein-associated DNA, including DNA packed in microvesicles, and DNase II digests the DNA from apoptotic cells (41, 42). Plasminogen, on the other hand, penetrates necrotic cells, accumulates in the cytoplasm and nucleus, and activates plasmin by tissue-type or urokinase-type plasminogen activator. Plasmin degrades histone H1 and facilitates internucleosomal DNA cleavage by DNase1 (43).

During NETosis, a substantial amount of serine proteases (e.g., PR3, MPO, NE, cathepsin G) are released into the extracellular space increasing the detrimental effects. To oppose this, ceruloplasmin disrupts MPO and limits MPO-dependent ROS generation, and alpha1-antitrypsin inhibits NE, PR3, and cathepsin G by forming complexes with them (44, 45).

## 3 The pathological roles of NETosis

### 3.1 Autoimmunity

While the beneficial effects of NETs have long been recognized, more recent studies have revealed that NETs also play a role in various pathological conditions, some of which may not be beneficial. The possibility that NETs may be involved in autoimmune diseases was initially suggested in 2004 by Brinkmann et al., leading to an increase in research on the subject (4). The role of NETosis in systemic lupus erythematosus (SLE), rheumatoid arthritis (RA), and ANCA-associated vasculitis (AAV) is well known today, but authors suggest that NETosis may also play a crucial role in other autoimmune and autoinflammatory conditions, such as type 1 diabetes mellitus, inflammatory bowel disease, gout, antiphospholipid syndrome, and in other rheumatic diseases (46–50).

Autoimmune diseases arise from the combination of genetic and environmental factors. NETs can be involved in breaking immune tolerance and triggering autoimmunity in a variety of pathways. After the initiation of an environmental factor, the propagation phase is characterized by inflammation and tissue damage. NETs contribute to propagation by epitope spreading and NET-forming cellular components (such as histones, DNA, and granular proteins) can serve as autoantigens for later antibody production.

Furthermore, NETs interplay with the adaptive immune system. LL-37-DNA complexes enhance autoantibody production on B-cells via TLR9 (51). Activated neutrophils release B cell-activating factor (BAFF) which upregulates the CD21 and CD19 co-receptor expression and prolongs the B-cell survival by decreasing proapoptotic proteins (52). The NET-derived immunoglobulins bind to the FcγR of the neutrophils and initiate further NETosis, creating a vicious circle (53). NETs prime CD4+ T-cells directly on T-cell receptors with a lower threshold, thus T-cell response is increased upon suboptimal NET component stimulation. On the other hand, T-cell response is also elicited in a dendritic cell-mediated manner. NETs induce costimulatory CD80 and CD86 on dendritic cells which interacts with T-cell CD28 for activation and survival, as well as interleukin (IL) production.

Interferon overproduction is a hallmark in the pathogenesis of several autoimmune disorders. Plasmacytoid dendritic cells are the primary interferon-producing cells, and they can be activated by NET-induced LL-37, HMGB1 (high mobility group box 1 protein), and DNA via TLR9 (54). NETs also mediate the activation of caspase-1, leading to inflammasome activation via the NLRP3 (NOD-like receptor family, pyrin domain containing) in macrophages. Inflammasome activation results in IL-18 and -1β secretion which further promotes NET formation (55).

Autoimmunity also occurs in case of failure to remove apoptotic and NETotic cells. As an example, NADPH-oxidase or PAD4 knock-out mice develop lupus-like disorders which contradict the studies that suggested improvement in the case of administration of NADPH-oxidase or PAD4 inhibitors in NET-related autoimmune diseases (56). Both enzymes are required for cellular debris removal by macrophages and the total absence of them exacerbates autoimmunity instead of ameliorating it (57). Furthermore, DNA accumulation as a result of the lack of DNase activity leads to prolonged inflammation and the presence of NET autoantigens. In susceptible individuals self-tolerance breaks, and an autoimmune response develops (41).

### 3.2 Autoinflammation

Based on the current classification, autoimmune and autoinflammatory diseases are positioned at opposite ends of a spectrum. In autoinflammatory diseases, immune tolerance remains intact and the pathomechanism is not driven by autoantigen-autoantibody interactions. Local and external factors, such as infections, mechanical damage, or temperature effects have a significant impact in triggering the disease, leading to innate immune responses and tissue damage (58, 59). These pathologies can be classified based on their genetic origin, distinguishing between monogenic and polygenic diseases, where the NF-κB, the NLRP3 inflammasome, and the IL-1β pathway play an important role in the signalization (60, 61).

Based on Matzinger’s “danger-theory”, in autoinflammatory diseases damage-associated molecular patterns (DAMPs) and pathogene-associated molecular patterns (PAMPs) serve as trigger factors for the innate immune system (62). Neutrophils engage in the process, however, the exact role of NETosis is still elusive. The various immunogenic proinflammatory factors released during NETosis can maintain sterile inflammation as an amplification loop, partly by stimulating the innate immune system to further recruitment and cytokine production by netting DNA, histone, and antimicrobial peptides; and partly via the activation of NLRP3-caspase 1 inflammasome system and IL-1β production (63, 64). matrix metalloproteinase (MMP)-mediated endothelial injury is a direct tissue-damaging consequence of histones and LL-37 (65). Prolonged IL-1β and IL-18 production can trigger T-cell differentiation and IL-18 can induce T-helper 1 and B cells. Accordingly, in some immunological cases, we can speak of autoinflammatory-autoimmune diseases of mixed etiology (like juvenile idiopathic arthritis, rheumatoid arthritis, Behcet’s disease, or adult-onset Still syndrome) (59, 66).

### 3.3 Thrombosis and atherosclerosis

Arterial and venous thrombotic and thromboembolic events can also occur via NETosis. NETs can serve as a scaffold for platelets to aggregate (10). Local hypoxia, NET compounds stimulate the endothelium further to release procoagulant factors and promote thrombus and NET formation. Additionally, tissue factor (TF) is released along with DNA, activating the extrinsic coagulation cascade pathway and increasing the risk of blood clots in the arterial and venous systems (67, 68). Along with the immunogenic effects, NETs also significantly contribute to vascular injury. Cytokines and extracellular histones damage endothelial cells directly as well as promote endothelial-mesenchymal transition (69). The NET-compound MMP-9 activates endothelial MMP-2 leading to endothelial cell death and augmenting the activity of collagenolysis, while interferons inhibit the endothelial progenitor’s differentiation (65, 70). NET proteins also modify the high-density lipoprotein (HDL) via oxidation, stirring it into a proatherogenic direction (71). Endothelial cells have limited capacity to take up the remnants of NETs, and persistent exposure to them provokes vascular leakage by demolishing intercellular junction proteins (69). Ultimately, these changes result in compromised endothelial function and vascular damage. Macrophages are recruited to remove NETs and promote plaque formation (11). Taken together, all these alterations lead to atherosclerotic diseases and an augmented potential to rupture and thrombus formation.

### 3.4 Tumorigenesis

The cancerogenic and tumorigenic roles of NETosis were observed as well (72). NETs can be found in large quantities around the tumorous microenvironment. NETs abolish tumor cells through their lytic enzymes and disrupt the extracellular matrix and intercellular connections, which can facilitate the migration of tumor cells (12, 73). NETs also assist the spread of cancer by capturing and helping metastatic cells to adhere to the tissue. They can also serve as a physical barrier, protecting tumor cells from cytotoxicity (73).

### 3.5 Kidney injury

**Figure 2:**
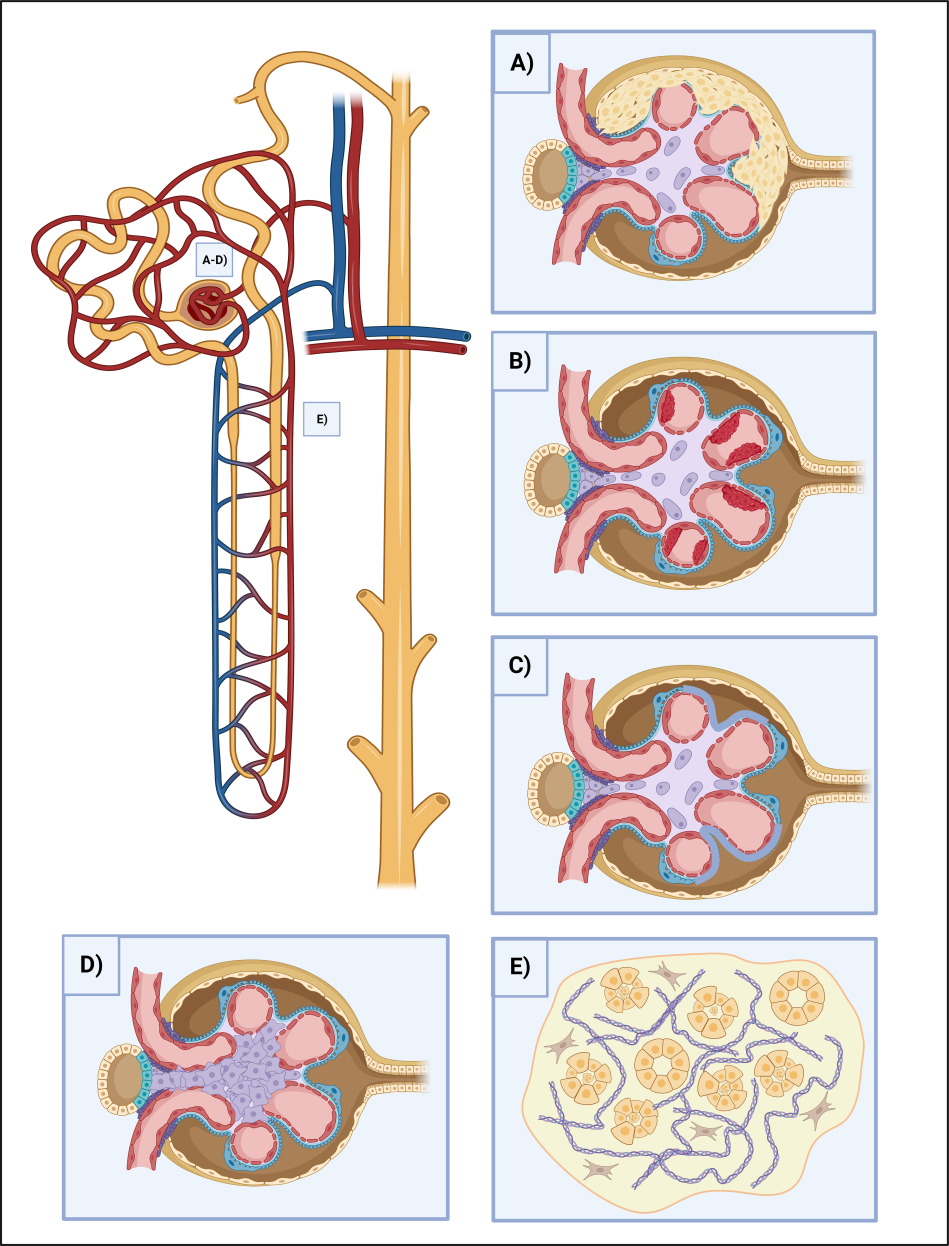
The effects of NETosis in kidney injury. **(A)** Crescent formation. Some NETosis products, such as histones and cytokines cause crescent formation by the induction of epithelial-mesenchymal and PEC progenitor proliferation. **(B)** Thrombosis. NETosis is heavily involved in clot formation. Besides NETs can serve as a scaffold for platelets, their contents damage the endothelium directly, oxidize HDL and stir it towards a proatherogenic direction, demolish the intracellular junctions and provoke vascular leakage, as well as stimulate the endothelium for further procoagulant factor (TF) release. **(C)** Podocyte foot process effacement. NETs disrupt the integrity of the slit-diaphragm by damaging the junctional proteins (podocin and nephrin), leading to the dysfunction of the glomerular filtration barrier, and causing proteinuria. **(D)** Mesangial proliferation. NETs cause ECM proliferation and the increment of mesangial cell count, leading to fibrosis. **(E)** Tubular injury. NETs play multiple roles in maintaining the destructive loop of tubular injury. NETosis-associated cytokine release causes tubuloepithelial cell necrosis, while histones have direct cytotoxic effects and help to prime neutrophils for further NETosis. ECM, extracellular matrix; HDL, high-density lipoprotein; NET, neutrophil extracellular traps; PEC, parietal epithelial cells; TF, tissue factor.

The effect of NETosis on renal cells is primarily conducted by the immune complexes and the consequently induced cytokines. However, the direct effect of NETosis on renal cells is not fully elucidated and only a limited number of studies are available. The releasing endogenous antigens from NETs are aggravating the inflammation since they act as DAMPs and further prime neutrophils and trigger NET production (37). Most importantly, extracellular histones convey significant cytotoxic effects with cytokines such as interferon and induce epithelial-mesenchymal and PEC (parietal epithelial cell) progenitor proliferation, thus crescent formation (74). Histones activate TLR-2,4 and NLRP3. NLRP3 mediates caspase-1 molecular complex (inflammasome) activation and promotes IL-1 and IL-18 cleavage to mature forms. Caspase-1 elicits pyroptosis in endothelial cells and it also contributes to platelet activation, aggregation, and microthrombus formation thus hemodynamic disturbances (75). NETs cause changes in the slit diaphragm-associated proteins, such as podocin and nephrin, provoking podocyte effacement and inducing consequential proteinuria. They induce podocyte cell hypertrophy, mitotic catastrophe, and eventually podocyte cell death as well (76). NETs hasten tubular epithelial cell apoptosis and further NET formation (74, 77) (**Figures 2A-E**).

## 4 Renal disorders with NET formation

One of the major objectives of this review was to summarize renal diseases in which NET formation is involved in the pathogenesis of the diseases. In the following paragraphs, we elaborate on these renal disorders.

### 4.1 SLE and lupus nephritis

**Figure 3:**
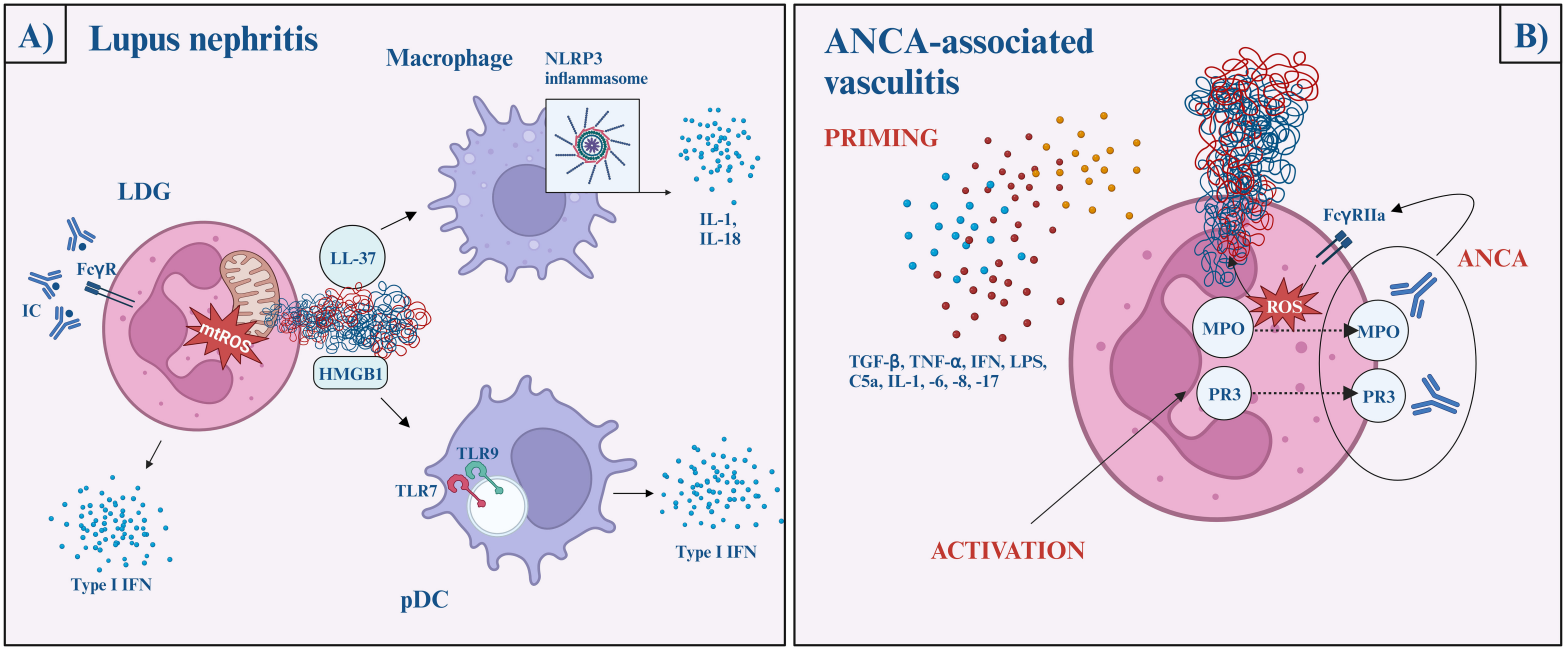
The role of NETs in lupus nephritis **(A)** and ANCA-associated vasculitis **(B)**. Lupus nephritis **(A)** Immune complexes activate neutrophils via the FcγR pathway and induce mitochondria-dependent NETosis. The NET-content (oxidized mtDNA, LL-37, and HMGB1-DNA complexes) later activates plasmacytoid dendritic cells via TLR7 or TLR9 and initiate type I interferon production, while macrophages are activated via the NLRP3 inflammasome pathway and facilitate IL-1 and IL-18 secretion. ANCA-associated vasculitis **(B)** Neutrophil priming occurs in response to various cytokines (TGFβ, TNFα, IFN, LPS, C5a, IL-1, -6, -8, -17), which reduces the neutrophil activation threshold. Then, MPO and PR3 are released onto the cell surface, where ANCA crossbinds them to neutrophil FcγRIIa and induces uncontrolled ROS and lytic enzyme bursts. After the burst, MPO and NE migrate to the nucleus, where NETosis begins. ANCA, anti-neutrophil cytoplasmic antibodies; AAV, ANCA-associated vasculitis; C5, complement factor 5; FcγR, fragment crystalline gamma receptor; HMGB1, high mobility group box 1; IFN, interferon; IL, interleukin; LDG, low-density granulocytes; LPS, lipopolysaccharide; MPO, myeloperoxidase; mtDNA, mitochondrial DNA; NE, neutrophil elastase; NLRP3, NLR family pyrin domain containing 3; pDC, plasmacytoid dendritic cell; PR3, proteinase 3; ROS, reactive oxygen species; TGF-β, transforming growth factor beta; TLR, Toll-like receptor; TNF-α, tumor necrosis factor alpha.

SLE is a multiorgan autoimmune disease characterized by the dysregulation of both the innate and adaptive immune systems, leading to inflammation and severe tissue damage across the body. About 30-50% of SLE patients develop lupus nephritis, which is a severe complication with proteinuria, kidney function loss, and increasing risk of mortality. 10-30% of the patients will progress to end-stage renal disease in 5 years. Impaired tolerance, aberrant response to self-antigens, and type I interferons are considered crucial in SLE development and pathogenesis, in which NETosis plays a critical role (4) (**Figure 3A**).

Low-density granulocytes (LDGs) are highly granular pathogenic granulocytes displayed in a high concentration in SLE patients. These neutrophils can be characterized by their ability to produce high amounts of proinflammatory cytokines, including type I interferons, and their increased propensity to undergo spontaneous NETosis (70, 78). Moreover, LDGs have a hyperability to produce mitochondrial ROS, which - as mentioned above - is adequate to generate NETs even in the lack of NADPH-oxidase and further stimulates interferon gene transcription (79).

This process is perpetuated by nucleic-acid-containing immune complexes. They activate neutrophils via FcγR pathways and induce mitochondria-dependent NETosis (79, 80). It is widely known that ultraviolet (UV) radiation exacerbates the pre-existing lupus disease. UV radiation induces NETosis wavelength- and dose-dependently. Penetrating the epidermis, UV-A and blue light induce ROS-production and subsequent MPO- and NE-dependent NETosis (81). UV-C radiation triggers mtROS generation, mtDNA decondensation, and caspase 3 cleavage, hence, features of both apoptosis and NETosis. Nevertheless, it is important to bear in mind that UV-C does not have biological importance since it is completely absorbed by the ozone layer (82)

On the other hand, oxidized mtDNA, exposed LL-37, and HMGB1-DNA complexes activate plasmacytoid dendritic cells via TLR9/TLR7, as well as endocytosed nucleic acid-autoantibody complexes can initiate type I interferon production, contributing to the prominent interferon signature in SLE (69, 83, 84). LL-37 and other NET proteins activate the NLRP3 inflammasome in macrophages, increasing the IL-1 and IL-18 secretion. As positive feedback, IL-18 and interferon α further stimulate the process of NET formation (2).

To conclude, NET formation in SLE is mostly NADPH-oxidase independent and induced primarily by immune complexes through FcγR signaling (85).

The accumulation of NETs is a common phenomenon in SLE and increases the exposure of nucleic acids and proteins to B-cells and plasmacytoid dendritic cells to generate high levels of interferons. There are three known ways in which the clearance of NETs may be impaired: 1) mutations and polymorphisms of DNase I, which lead to inadequate enzyme function, 2) inhibition of DNase by autoantibodies, and 3) the presence of anti-NET antibodies, that hide the binding sites from DNase I (86–89). In the latter form, autoantibodies recruit complement; C1q binds to DNA and prevents DNase I from degrading NETs (90). Furthermore, oxidized DNA is more resilient to DNase degradation (91). In summary, the altered clearance results in a longer exposure time to NETs and autoantigens, therefore it correlates with the severity of the disease. In parallel with this, the amount of DNase I in the kidney and urine decreases as lupus nephritis progresses (92).

### 4.2 Anti-neutrophil cytoplasmic antibody-associated vasculitis

Anti-neutrophil cytoplasmic antibody (ANCA)-associated vasculitis (AAV) is a necrotizing small vessel vasculitis characterized by the presence of circulating ANCAs. Based on the circulating antibodies and clinical manifestations, three main forms of AAV are distinguished: microscopic polyangiitis (MPA), granulomatosis with polyangiitis (GPA), and eosinophil granulomatosis with polyangiitis (EGPA). Renal involvement with nephritis syndrome (kidney function loss, hematuria, high blood pressure) is a common manifestation of AAVs occurring in most cases of MPA and frequently in GPA, however, it is rare in EGPA (93). The diagnostic hallmark of AAV is antibodies targeted against granular proteins of neutrophils: MPO and PR3.

Neutrophil priming is presumed to be the key step in NET formation in AAV. Priming is a process in which the neutrophil response is enhanced by an activating stimulus. Priming undergoes with the help of proinflammatory cytokines (such as transforming growth factor-beta, tumor necrosis factor α, interferon-α, -γ, IL-1, -6, -8, -17) complements (C5a), and LPS. After neutrophil activation, MPO and PR3 exteriorize on the cell surface. ANCA crossbinds these antigens with neutrophil FcγRIIa and induces uncontrolled ROS and lytic enzyme bursts. After the burst, MPO and NE migrate to the nucleus and NETosis begins and run its course in an NADPH-oxidase-dependent pathway, via the lytic NETosis. This is a pivotal difference compared to SLE (94, 95).

Although ANCA stimulation is suggested to be a key to NET formation, recent studies highlight a possibility of an ANCA-independent manner of NETosis. NETosis still occurs when ANCA IgG and IgA are depleted, or when the C5a receptor is inhibited. It does not correlate with serum tumor necrosis factor α (TNF-α), IL-6, -8 (priming cytokines), or conventional inflammatory markers like CRP or erythrocyte sedimentation rate. In fact, the level of NETosis is higher in ANCA-negative AAV patients. Additionally, even though relapse in AAV is commonly triggered by a concurrent infection, NET formation in AAV patients was lower during severe infection than in patients with relapsing AAV. To sum up, the exact mechanism of NET formation in AAV patients remains unknown and the increased level of NETs is linked to autoimmunity and clinical disease activity rather than concomitant infection (96) (**Figure 3B**).

### 4.3 Diabetic nephropathy

Diabetes can affect kidney function through several mechanisms. Classic diabetic nephropathy is considered a microvascular complication of diabetes, which will lead to proteinuria and glomerulosclerosis. Although diabetes is closely associated with inflammation and oxidative stress, the role of neutrophils in this process is almost neglected. Increased glucose level upregulates PKC activity and induces NADPH-oxidase overstimulation and oxidative burst regardless of the type of diabetes. As described above, oxidative burst is a pivotal step in NET formation. Inflammatory cytokines and free fatty acids inhibit insulin signaling by phosphorylation of inhibitors of nuclear factor kappa-B kinase (IKKβ) and c-Jun N-terminal kinase 1 (JNK1), which are also inflammatory pathway mediators, and induce NFκβ (nuclear factor kappa-light-chain-enhancer of activated B cells) translocation to the nucleus, resulting in various proinflammatory gene activation which is necessary for the priming process (97). Moreover, high extracellular glucose polarizes macrophages to a proinflammatory M1 phenotype. Interacting with NETs, M1-macrophages not only exacerbate the proinflammatory response but also go under apoptosis and release extracellular DNA. This mechanism is initiated specifically by NETs and contributes to an augmented load of free DNA and the progression of the disease (36). Although the underlying pathomechanisms in the development of diabetic kidney disease are complex and go beyond the scope of this review, it is clear that NETosis is noticeably involved in the process.

### 4.4 Acute tubular necrosis

Acute tubular necrosis (ATN) is the most common form of acute kidney injury (AKI), and it is characterized by the destruction of the tubular epithelial cells leading to a rapid kidney function loss with oliguria or anuria. It may occur as a result of ischemic or toxic impacts. Acute tubular necrosis is accompanied by a massive inflammatory response including recruitment, activation of immune cells, and increased proinflammatory cytokine production. Renal cell necrosis releases necrotic cell debris which is introduced as DAMPs. The innate immune system and especially neutrophils are the major responder and effector cells in AKI and play a role in the crescendo-type inflammation (98). DAMPs, such as histones generate a secondary autoimmune amplification loop by further priming neutrophils, activating them to form NETs, further deteriorating the kidney injury and exposing even more endogenous epitopes (77). Uromodulin is a constitutively secreted protein by epithelial cells in the thick ascending limb and the distal tubule into the tubular lumen. Uromodulin binds and aggregates cytokines in the lumen and stays inert inside the luminal compartment. However, in case of tubular damage, uromodulin compiles in the interstitium as crystal-like structures and they can activate antigen-presenting cells and elicit the NLRP3-inflammasome-caspase-1 pathway (99). In the late phase of the injury, macrophages infiltrate the injured tissue and in the proinflammatory environment, they undergo an M1-phenotype shift contributing to necroinflammation. Contrary to necrosis, apoptosis is a balanced and regulated physiological mechanism that halts the exaggerated inflammatory process. Apoptosis of the recruited immune cells polarizes phagocytes to M2 phenotype and favors an anti-inflammatory milieu by secreting Il-10, TGF-β, and growth factors. This environment promotes not only suspending the inflammatory vicious circle but also contributes to the regeneration of epithelial and vascular healing and fibrosis (37).

### 4.5 Anti-glomerular basement membrane disease

Anti-glomerular basement membrane (GBM) disease is a rare autoimmune disorder in which antibodies are produced against type IV collagen and presents with rapidly progressive glomerulonephritis and alveolar hemorrhage (100). Anti-GBM IgG binds FcγRIIa in a shear-force-dependent manner. FcγRIIa binding causes F-actin polymerization via the Abl/Src mediated pathway. Their interaction and actin polymerization leads to an endothelial CD18 integrin (Mac-1) activation, and integration-mediated adhesion takes over selectin-mediated rolling. Thus, neutrophil attachment to the endothelial cells in the capillaries is sustained. Eventually, FcγR engagement triggers intravascular ROS, protease, and NET generation (101, 102). This mechanism with the extensive NET formation and the consequent exaggerating necroinflammation explains the substantial renal damage in anti-GBM glomerulonephritis which not only confines to the glomeruli but also affects the interstitium and the tubules.

### 4.6 Hemolytic uremic syndrome

Hemolytic uremic syndrome (HUS) is a thrombotic microangiopathy caused by enterohemorrhagic Shiga toxin (Stx) - producing bacteria (Shigella dysenteriae or enterohemorrhagic Escherichia coli). The syndrome is characterized by thrombocytopenia, hemolytic anemia, and acute renal failure. It is one of the main causes of acute kidney injury in children and has no specific treatment.

In hemolytic uremic syndrome, neutrophils are prone to undergo NETosis. There are several mechanisms behind it: 1) Shiga toxin is believed to be a potent neutrophil activator and NET inducer. 2) Uric acid is released from the injured cells in a great quantity, crystallizes, and triggers NET formation. 3) Higher dose of Stx induces NETosis in a ROS-dependent manner. 4) Stx enhances P-selectin expression on LPS-treated platelets and promotes neutrophil activation and aggregation (102).

Furthermore, exaggerated endothelial damage and thrombosis formation can be observed in HUS. Besides the excessive release of endothelial toxic NET contents, LPS, and Stx-treated platelets also contribute to endothelial damage (102). The role of neutrophil extracellular traps in atypical hemolytic uremic syndrome and thrombotic microangiopathies, however, is unclear.

### 4.7 Autoinflammatory diseases with renal involvement

Gout is an autoinflammatory condition characterized by monosodium urate (MSU) crystal deposition in the joints and the kidneys. High uric acid concentration is also a risk for nephrolithiasis.

MSUs are taken up by phagocytes and either directly damage the cell membrane causing necrosis and necrotic debris release leading to inflammatory cell recruitment, cytokine and chemokine production, or activating the NLRP3-caspase-1 inflammasome system resulting IL-1β release (50, 103). Recruited neutrophils undergo NADPH-dependent *lytic* NETosis, partly via direct activation by MSUs and partly by the inflammatory mediators (104). Uniquely, NETosis has a dual role in gout. Besides perpetuating inflammation, NETosis also limits it by engulfing MSUs preventing them from further phagocyte activation. MSU-stimulated neutrophils form aggregated NETs, which can proteolytically degrade and inactivate cytokines and chemokines stopping gout attack and resolving inflammation (105, 106).

Familial Mediterranean Fever (FMF) is a monogenic autoinflammatory disease, with clinical characteristics of fever attacks, joint pain, and skin rashes. During fever attacks, neutrophils produce large amounts of NETs which perpetuate inflammation via directly derived IL-1β and by inducing polymorphonuclear cells for further IL-1β production. However, NETs also have a self-limiting, therefore anti-inflammatory effect, as they can prevent further NETosis and stop the fever attack (107, 108). The most common kidney damage caused by FMF is amyloidosis, but IgA nephropathy and mesangioproliferative glomerulonephritis were also described (109).

## 5 Potential therapeutic targets in NETosis

Since the signalization of NETosis is sprawling, the number of potential therapeutic targets is extensive. Nevertheless, NET inhibition in renal disorders is not exhausted yet, most agents are yet to be trialed in kidney diseases. Here we list the most examined and most promising drug candidates in clinical practice.

### 5.1 Disease-modifying antirheumatic drugs

#### 5.1.1 Conventional synthetic DMARDs

##### 5.1.1.1 Recombinant DNase

DNase is responsible for the disassembly of the NET-related nucleoproteins and facilitates the clearance by macrophages. Recombinant DNase eliminates the NETosis products and delays the development of antibodies against them. Nevertheless, recombinant DNase I treatment is controversial. It does have a good efficacy to reduce NETs in COVID-triggered acute respiratory distress syndrome via inhaled route or when administered intravenously in lupus nephritis patients and anti-MPO ANCA mouse model, however, intravenous or subcutaneous administration failed to achieve sufficient bioactive serum concentration in humans (94, 110, 111). Recombinant DNase I is inactivated rapidly by G actin by forming a complex with it. To overcome this limitation, adeno-associated virus vectors were used in a lupus-prone mice (NZBWF1) model. This way, DNaseI activity was maintained for more than 6 months, and renal neutrophils, NETs, IgG, and C3 were significantly reduced. However, this way of rhDNase administration still did not extend lifespan and preserved renal function (112).

##### 5.1.1.2 Toll-like receptor inhibitors

Hydroxychloroquine (HCQ) and chloroquine (CQ) are anti-malarial drugs frequently used in autoimmune diseases, due to their immunomodulatory effects. They inhibit NET formation by antagonizing TLR-3, - 7, -8, -9 (113). Furthermore, hydroxychloroquine also has an inhibitory effect on cyclic GMP-AMP synthase (cGAS), a cytosolic DNA sensor responsible for type I interferon response. Taken together, the downstream proinflammatory cytokine production will be suppressed (114). Chloroquine has been shown to reduce autophagy and increase the pH in lysosomes, impeding antigen presentation on MHC class II molecules for CD4+ T-cells. Additionally, hydroxychloroquine is suggested to inhibit PAD4 as well (115). Utilizing these pharmacological effects, hydroxychloroquine and chloroquine are demonstrated to reduce SLE and especially lupus nephritis flares, help to maintain remission and delay the onset of complications (116, 117). Hydroxychloroquine attenuated anti-GBM nephritis in WKY rats as well which was attributed to the suppression of JNK/p38 MAPK phosphorylation (118).

The dual TLR 7/8 inhibitor enpatoran was well-tolerated in a phase 1 study in healthy participants and a current WILLOW study is evaluating its efficacy in SLE patients, however, patients with active lupus nephritis have been excluded (NCT05162586) (119). TLR inhibition with GIT27 (VGX-1027) mitigated kidney injury in a diabetic experimental mouse model. GIT27 targets primarily TLR4 but also interferes with TLR2/6 signaling pathways in macrophages which results in a lower profibrotic and proinflammatory profile, lower albuminuria, and mesangial expansion (120). TAK-242 and eritoran are selective TLR4 inhibitors while NI-0101 is a humanized monoclonal antibody against TLR4 (121). TAK-242 ameliorated rhabdomyolysis, acetaminophen, and contrast-induced acute kidney injury (122–124). TAK-242 also reduced serum creatinine and blood urea nitrogen concentration in sepsis-mediated kidney injury (125). Nevertheless, it is important to bear in mind that TLR4 activation and the subsequent proinflammatory response are crucial for bacterial clearance in sepsis.

Taken together, Toll-like receptor inhibitors are promising therapeutic agents in NETosis-related diseases, however, in previous clinical trials none has yet been proven clinically useful (126, 127).

##### 5.1.1.3 Calcineurin inhibitors

Cyclosporin, tacrolimus, and voclosporin are calcineurin pathway inhibitors widely used in organ transplants and autoimmune disorders. They bind cytophilin and downregulate the transcription factor nuclear factor of activated T cells (NFAT) and inhibit the calcineurin pathway. Studies proved a significant influence on neutrophils suppressing chemokinesis, adhesion, angiotensin II response, phagocytic activity reduction, and reducing the extent of IL-8 induced NETosis (128). Calcineurin inhibitors also block T-cell overactivation and podocyte alteration. This results in proinflammatory mediator release from T-cells and cytoskeleton stabilization and apoptosis inhibition in podocytes emphasizing their powerful roles in autoimmune renal disorders (129). Although calcineurin inhibitors are not recommended as first-line therapy in lupus nephritis, cyclosporin, and tacrolimus can be used in combination with mycophenolate-mofetil in nephrotic range proteinuria. The combination of voclosporin and mycophenolate-mofetil was associated with a higher rate of complete remission at 6 months as compared with mycophenolate alone (130).

Tacrolimus improved albuminuria and tubulointerstitial damage, and ameliorated macrophage infiltration and proinflammatory cytokine expression in diabetic db/db mice (131). Tacrolimus was proved to recover the nephrin expression, thus maintaining the structural and functional integrity of podocytes in diabetic Sprague-Dawley rats (132).

##### 5.1.1.4 Colchicine

Colchicine is an alkaloid derivative that has pleiotropic anti-inflammatory effects. It attaches to soluble tubulin in an almost irreversible manner and inhibits the elongation of the microtubules. In higher concentrations, it also promotes microtubule depolymerization, alters endothelial E-selectin distribution, promotes L-selectin shedding on neutrophils, inhibits superoxide production and NLRP3. Colchicine inhibits immune response by dampening neutrophil chemotaxis, recruitment, adhesion, inflammasome activation, and NETosis as well. Colchicine blocks inflammatory response via regulating NF-kB and caspase-1 (133). The advantageous effects of colchicine in NETosis were proved in a small subset of patients, however, its potential renal toxicity in comprised kidney function limits its use in possible utilization in NET-related kidney disorders (134–136). Colchicine was studied in a randomized controlled double-blind clinical trial in diabetic nephropathy where colchicine decreased neutrophil-related chronic inflammation; however, it did not lower creatinine, urinary albumin/creatinine ratio and did not prevent overt nephropathy either (137).

##### 5.1.1.5 PAD4-inhibitors

Inhibition of PAD4 via Chlor-amidine (Cl-amidine) irreversibly blocks the active sites of the enzyme via covalent modification (138). GSK484 and GSK199 are selective and reversible inhibitors that bind to PAD4 with a high affinity (139).

Cl-amidine significantly inhibits NET formation in NZM model of murine lupus, reduces complement consumption, glomerular IgG deposition, and although not significantly but reduces albuminuria. Cl-amidine administration to NZM mice improved endothelium-dependent vasorelaxation and delayed arterial thrombosis which was partly attributed to the reduction of NET formation (140). Following PAD inhibition, immune complex deposition, interstitial inflammation, and urine albumin/creatinine ratio decreased in MRL/lpr lupus nephritis model as well (141, 142).

Cl-amidine attenuated kidney injury in a rabbit model of LPS-induced acute kidney injury. Cl-amidine decreased histopathologic signs of acute kidney injury, as well as elevated creatinine and blood urea nitrogen (143). GSK484 mitigated renal ischemia-reperfusion injury in C57BL/6 mice by reducing NETosis (144).

Although PAD4 inhibition proved to be effective in the suppression of NETosis in several *in vitro* and *in vivo* animal models, clinical trials have not been conducted yet. On the other hand, inhibition strategy with PAD4 is a double-edged sword since it inhibits essential NET formation as well.

#### 5.1.2 Targeted synthetic DMARDs

##### 5.1.2.1 Tyrosine kinase and JAK-inhibitors

Bosutinib is a selective dual Abl/Src inhibitor used in chronic myeloid leukemia (CML) to inhibit the Bcr-Abl fusion protein. However, based on this function, the utilization of bosutinib might be reevaluated and repurposed. It proved to be beneficial in anti-GBM glomerulonephritis since it averted an early step of inflammation. Bosutinib reduced the FcγRIIa-mediated adhesion, neutrophil recruitment, and NET formation in an *in vitro* experiment (101). Another selective Abl/Src inhibitor dasatinib significantly reduced neutrophil and macrophage influx in skin wounds, thus accelerating healing and reducing scarring (145). On the other hand, it is important to note that the multi-tyrosine kinase inhibitor ponatinib increases NET production which contributes to vascular toxicity in CML patients (146).

Neutrophil Bruton’s tyrosine kinase (Btk) mediates reactive oxygen species generation via TLR4-signals, and integrin-mediated recruitment and activation (147–149). Btk inhibitors reduced proteinuria and improved glomerular histopathology in lupus-prone animal models (150–153). Elsubrutinib is currently under the scope for efficacy in SLE patients including lupus nephritis alone or in combination with upadacitinib compared to placebo (NCT03978520). Nevertheless, previous studies with Btk inhibitors (fenebrutinib, evobrutinib) did not bring the expected results in SLE patients, though patients with active LN were excluded (154, 155).

Spleen tyrosine kinase (Syk) is an adapter protein that is responsible for phosphorylating several other adapter proteins to propagate signalization cascades resulting in cytokine/chemokine release via NF-κB, actin cytoskeleton remodeling, proliferation, and differentiation via the MAPK pathway and also for survival via the NFAT (nuclear factor of activated T cells) pathway (156). Syk is activated by phosphorylation upon ANCA binding to FcγRIIa in TNF-α–primed neutrophils and induces respiratory burst. Syk is also an essential regulator in B-cell receptor-mediated signalization and enhanced function is related to the pathogenesis of autoimmune disease and B-cell leukemias and lymphomas as well (157). Fostamatinib is a highly selective Syk inhibitor, and it was proven to improve MPO-ANCA glomerulonephritis in a pre-clinical ANCA vasculitis model in Wistar Kyoto rats (158). Fostamatinib delayed renal progression in NZB/NZW mice in lupus nephritis model and anti-GBM glomerulonephritis in Sprague-Dawley and WKY rats (159–161). Fostamatinib has not undergone a clinical investigation yet. A trial had commenced years ago that targeted SLE patients, but it was withdrawn before it could have been started (NCT00752999).

Downstream inhibition of the JAK/STAT pathway with tofacitinib is proven to lower NET formation (162). Tofacitinib decreased proteinuria, reduced mesangial cell proliferation, and glomerular IgG deposition. Furthermore, after tofacitinib administration in MRL/lpr mice, TGF-β receptor 1 was reduced in renal tissues, thus tofacitinib alleviated renal fibrosis in MRL/lpr mice model (163). Other JAK inhibitors, like baricitinib, peficitinib, upadacitinib and filgotinib are approved as disease-modifying antirheumatic drugs (164).

Baricitinib was a promising agent in SLE, however, it failed to decrease the SLEDAI (Systemic Lupus Erythematosus Disease Activity Index) or the BILAG class in SLE-BRAVE II study (165). Although lupus nephritis patients were excluded from this trial, other ongoing studies are taking chances on baricitinib and LN patients (NCT05686746). Brepocitinib and upadacitinib are other JAK inhibitors that are currently subjects to SLE studies, however, renal involvement with brepocitinib is among the exclusion criteria (NCT03845517, NCT03978520). The phase II result of the upadacitinib study is expected to be published soon (NCT03978520).

An abundance of evidence showed the progression of diabetic kidney disease is boosted by inflammation that is mediated by the JAK-STAT pathway. Baricitinib decreased the urinary albumin/creatinine ratio from baseline at 6 months compared to placebo (166). This finding is supported by two previous trials in which patients with diabetic nephropathy received monocyte chemoattractant protein 1 (MCP1) inhibitors (167, 168). Ruxolitinib contributed to lower proinflammatory cytokine levels and regulated podocyte autophagy in diabetic kidney disease (169, 170). Although it has been proven that ruxolitinib blunts NET formation in chronic myeloproliferative neoplasms and its NET inhibiting role has not been investigated in renal disorders, it is suspected that NET suppression also contributes to the amelioration of diabetic nephropathy (171, 172).

Tofacitinib attenuated LPS-induced acute kidney injury by inducing intrarenal proinflammatory cytokine production and reducing oxidative stress (173). The same effect was reached when C-X-C Motif Chemokine Receptor 1 and 2 (CXCR1/2) was inhibited by the chemokine C-X-C motif ligand 8 (CXCL8) antagonist, G31P (174).

#### 5.1.3 Biological DMARDs

##### 5.1.3.1 C5-inhibitors

Priming via C5a-C5a receptor interaction is crucial in NET formation. The C5a receptor antagonist, avacopan, and the anti-C5a monoclonal antibody, eculizumab have been both proven to reduce NET formation (2). The orally administered avacopan was approved as an adjunct therapy for microscopic polyangiitis and granulomatosis with polyangiitis which permits a lower glucocorticoid exposure and reduces the glucocorticoid-related toxicity, thus improving patient quality of life (175). Eculizumab is an anti-C5 monoclonal antibody that inhibits complement-mediated thrombotic microangiopathy and significantly improves renal function in patients with atypical hemolytic-uremic syndrome (176). Eculizumab is a therapeutic option in individuals with lupus nephritis who developed subsequent thrombotic microangiopathy and patients with refractory lupus nephritis might benefit from supplementary eculizumab therapy as well (177). Moreover, in a case report eculizumab was used as rescue therapy and improved renal outcome in two patients with anti-GBM glomerulonephritis (178).

##### 5.1.3.2 Interferon pathway inhibitors

As previously described, type I interferons elicit numerous immunological effects, including the assistance of NET formation. Therefore, interferon blockade is an ideal pharmacological approach to decrease NET production. Sifalimumab and rontalizumab are monoclonal antibodies against interferon α and aid their neutralization. Both of them underwent trials in SLE patients and reached superiority above placebo, however, in these trials, patients with renal involvement were excluded (179, 180). Anifrolumab is a human immunoglobulin targeted against subunit 1 of the interferon α receptor, conveying a general inhibition signal (181). Anifrolumab decreased the BILAG (British Isles Lupus Assessment Group)-based Composite Lupus Assessment (BICLA) index in SLE patients in the TULIP study, however, this study also excluded lupus nephritis patients (182). Nevertheless, the TULIP-LN study evaluated the efficacy of anifrolumab in renal involvement. Unfortunately, the primary endpoint of the study was not met, there was no significant improvement in the 24-hour protein/creatinine ratio from baseline to 52 weeks in anifrolumab-treated patients versus placebo. Authors explain the results by the suboptimal dosing and the increased clearance of anifrolumab associated with proteinuria (183).

##### 5.1.3.3 Depletion of B-lymphocytes

B-cells contribute to NET formation mainly via antibody production and immune complexes; however, they are also potent sources of cytokines for priming. The primary B-lymphocyte depleting agents are rituximab (anti-CD20 monoclonal antibody) and belimumab (B-lymphocyte stimulator (BLyS)/BAFF inhibitor human monoclonal antibody). They are used mostly individually but sequential therapy also has its rationale: belimumab targets CD20+ and CD20- plasmablasts but spares the CD27+ memory cells, while rituximab reduces CD20+ cells effectively - including CD27+ memory cells- but does not affect CD20- plasmablast cells (184). Obinutuzumab is a new-generation anti-CD20 monoclonal antibody designed to overcome rituximab resistance. Obinutuzumab has a greater affinity for FcγRIII and employs different mechanisms of action. It evokes a greater direct B-cell death by a more potent antibody-dependent cellular toxicity and phagocytosis (185).

Rituximab used to be a promising agent in proliferative lupus nephritis, but the renal response rate was not superior over mycophenolate-mofetil and glucocorticoids at 1 year (LUNAR study) (186). Nevertheless, it may still be considered in persistent disease activity or in repeated flares (187). On the contrary, belimumab helped to reach renal response beside standard therapy (BLISS-LN study) so it was included in Kidney International Improving Global Outcomes (KDIGO) guidelines as the first on-label monoclonal antibody in SLE treatment (187, 188). The combination of belimumab and rituximab effectively reduced NET formation in human SLE by reducing the circulating immune complexes (189). An ongoing randomized controlled study (SynBioSe-2) is investigating the combination treatment protocol of belimumab followed by rituximab in lupus nephritis patients compared to prednisolone and mycophenolate-mofetil induction (NCT03747159). Nevertheless, an *in vitro* study contradicted this result and showed enhanced NETosis after Rituximab treatment (190). The NOBILITY trial was designed to evaluate the hypothesis that adding obinutuzumab to the standard glucocorticoid and mycophenolate-mofetil treatment improves the rate of complete renal remission compared to the treatment without obinutuzumab. Obinutuzumab add-on therapy was superior to placebo in proliferative lupus nephritis to reach complete renal response at 2 years (191).

The induction and maintenance protocol of ANCA-associated glomerulonephritis also involves rituximab. Beside glucocorticoids, the standard induction therapy is either cyclophosphamide and/or rituximab (187). Rituximab is also highly efficient in relapsing disease and anti-PR3 ANCA-positive patients resulted in a higher remission rate post-rituximab treatment (192, 193). Belimumab was studied as a maintenance agent, but it did not reduce the risk of relapse. However, utilizing the two agents’ additive synergistic effects, an ongoing study (COMBIVAS - NCT03967925) uses rituximab and belimumab combination therapy as induction in PR3 vasculitis. Obinutuzumab was reported to reach remission successfully in three cases of ANCA-associated vasculitis. An ongoing randomized, double-blind study (ObiVas) is currently recruiting participants for a phase II study to compare the efficacy of rituximab and obinutuzumab in AAV patients [ISRCTN13069630].

Rituximab use has been reported for the treatment of anti-GBM with various outcomes. Although B-cell depletion inhibition is not part of the KDIGO treatment guidelines and standard-of-care approaches in anti-GBM glomerulonephritis, rituximab may be initiated in cases of refractory to standard therapy or in relapsed disease (187, 194). Although anti-GBM antibody vanishes after rituximab therapy in the majority of cases, it cannot halt kidney injury or reverse dialysis dependency (194). However, the negative renal outcome is an inherent consequence of the rapid pathomechanism of anti-GBM glomerulonephritis not the inefficacy of immunosuppressive therapies.

##### 5.1.3.4 Interleukin antagonists

Tocilizumab is a humanized monoclonal antibody against the interleukin-6 receptor. It decreases endothelial dysfunction and oxidative stress and downregulates low-density granulocytes in rheumatoid arthritis.

Tocilizumab has already been proven effective in large-vessel vasculitis and a few reports also reported remission in microscopic polyangiitis with tocilizumab. SATELITE study is going to evaluate the efficacy in patients with granulomatosis with polyangiitis (NCT04871191).

Tocilizumab showed a promising clinical and serological response in SLE patients; however, patients with fulminant renal involvement were excluded. PF-04236921 is a fully-humanized monoclonal antibody against IL-6 that was not significantly different from placebo for reducing the disease activity (195).

Tocilizumab ameliorated proteinuria in streptozotocin-induced diabetic nephropathy rat model and attenuated histological changes and was also beneficial for podocytes in mice (196, 197). Tocilizumab mitigated kidney function deterioration in a case report, however, clinical trials have been not conducted yet (198).

Anakinra (human IL-1 receptor antagonist), canakinumab (human monoclonal antibody against IL-1β) and rilonacept (soluble decoy receptor) are used mostly in the treatment of autoinflammatory diseases (e.g., juvenile idiopathic arthritis, gout). *In vitro*, pre-treatment with anakinra successfully reduced NET formation in PMA-induced NETosis in a time- and dose-dependent manner (199). Anakinra was successfully used to partially inhibit NET production in experimental gout models. Both anakinra and canakinumab attenuated MSU-induced NETosis via altering NLRP3-inflammasome activation and IL-1β release (50, 200). Rilonacept is an IL-1α and IL-1β trap, which is approved in the treatment of cryopyrin-associated periodic syndromes and recurrent pericarditis (201), however, exact effect on NETs is not well described.

The anti-IL-18 monoclonal antibody GSK1070806 have been tested in renal transplant delayed graft function (202), obesity and type 2 diabetes (203), but was not proven effective. Recombinant human IL-18 binding protein, which neutralize IL-18 along with IL-37 had more success in experiments and clinical trials in psoriasis, rheumatoid arthritis, hemophagocytic lymphohistiocytosis, and adult onset Still disease (204). To date, there is no approved treatment targeting IL-18.

#### 5.1.4 Miscellaneous

##### 5.1.4.1 Antioxidants

Amino salicylates scavenge ROS superoxide and recover SOD activity, NF-*κ*B, MPO, and proinflammatory cytokine production. The inhibitory effect of 5-aminosalicylic acid and acetylsalicylic acid on NETosis was proved, making them a potentially ideal supplementary therapy in NET-related kidney disorders (205, 206).

N-acetylcysteine reduces NETosis by modulating ROS production (207).

N-acetylcysteine (NAC) slightly reduces albumin/creatinine ratio and suppresses renal fibrosis in diabetic nephropathy in diabetic rat models (208, 209). High-dose NAC decreased SLEDAI scores and BILAG classes significantly in a randomized double-blind clinical trial, though patients with acute flare threatening vital organs were excluded (210). Pre- and post-treatment with NAC in septic rats were protective against kidney injury while long-term administration of NAC worsened organ failure and did not improve albuminuria. As a matter of fact, high-dose NAC administration in patients with over 24-hour duration sepsis resulted in cardiac depression, hypotension, and worsened acute kidney injury (211–215). Interestingly, NAC employed protective effects when administered in short-term ischemia in Wistar rats. NAC treatment decreased renal vascular resistance, thus increased renal blood flow and prevented histopathological changes when ischemia was applied for 30 minutes compared to 45 minutes. This suggests that NAC treatment might have a therapeutic window to utilize the nephroprotective effect (216). NAC also failed to offer renal protection in patients with stage 3 chronic kidney disease at risk of contrast-induced nephropathy (217).

Besides being a potent antidiabetic drug, metformin has mTOR and consequential mtROS production inhibitory effects. During PMA- and/or Ca-induced NETosis, metformin effectively reduced NETosis via inhibition of NADPH-oxidase and the membrane translocation of PKC-βII (218).

Metformin improves renal function in MRL/lpr lupus-prone mice. It decreased the proinflammatory cytokines and the levels of anti-nuclear antibody and anti-double stranded DNA antibodies. Metformin administration also attenuated histopathological damages (219). A *post hoc* analysis demonstrated that metformin decreased the incidence of flares in SLE patients, though patients with renal impairment were excluded (220). A study aimed to evaluate the preventive effects of metformin in lupus nephritis (NCT04145687), however, the current status is unknown.

Metformin inhibits mitochondrial respiratory complex I, and reduces ATP synthesis, hence increases AMP/ATP and ADP/ATP ratios. It leads to the activation of adenosine-monophosphate-activated kinase (AMPK). AMPK inhibits hepatic gluconeogenesis, lowering glucose toxicity. Metformin alleviates the glucose- and advanced glycation product (AGE)-induced NF-κB activation, ROS production, and the subsequent proinflammatory cytokine production (221). By this, metformin does not only repress intrarenal inflammation but also proliferation and fibrosis (222).

Idebenone is a ubiquinone analog antioxidant agent with ROS scavenger properties, hence a mitochondrial ROS inhibitor. It defends membranes from lipid peroxidation and restores ATP production in the mitochondria (223). In MitoTEMPO study it was proved to inhibit spontaneous NET formation of low-density granulocytes and decrease disease activity in SLE (224).

##### 5.1.4.2 Vitamin D

Vitamin D is a fat-soluble secosteroid with pleiotropic function. The role in NETosis was shown by Handono et al. who studied SLE patients with hypovitaminosis D. Results showed that vitamin D3 substitution inhibited NETosis activation and decreased consequential endothelial damage, however, the pathomechanism remains unknown (225). Vitamin D was significantly lower in patients with lupus nephritis compared to patients with either active SLE without nephritis or inactive SLE (226). Another study demonstrated an inverse correlation between SLEDAI and serum 25-hydroxy-cholecalciferol and proved that higher levels of vitamin D were associated with a decrease in urine protein/creatinine ratio (227).

Although a negative correlation was observed between 25-hydroxy-cholecalciferol and Birmingham vasculitis activity score (BVAS) in AAV, no difference was revealed in 25-hydroxy-cholecalciferol levels among AAV patients with and without renal involvement (228).

A double-blind, randomized-controlled study demonstrated that the vitamin D analog paricalcitol decreased urinary albumin excretion significantly compared to placebo (229). Another randomized-controlled trial with type 2 diabetic patients showed a faster decline in estimated glomerular filtration rate in patients with 25-hydroxy-cholecalciferol deficiency (230).

Macrophage and lymphocyte infiltration in ischemia-induced kidney injury was proven to be significantly higher and the risk of acute kidney injury was 1.2- and 1.5-fold higher in patients with 25-hydroxy-cholecalciferol insufficiency and deficiency (231, 232). Although vitamin D has a pleiotropic effect in acute kidney injury, the participation of NETosis cannot be omitted either (231).

##### 5.1.4.3 Antibiotics

Azithromycin exerts a dose-dependent ROS inhibition, hence preventing the NET release. Gentamicin and chloramphenicol also decrease NETosis, the latter probably via decreasing MPO activity. Nevertheless, their efficacy was tested merely preclinically (233, 234).

##### 5.1.4.4 Gasdermin D inhibition

Inhibition of Gasdermin D effectively halts the cascade of inflammatory molecule release in NETosis. Disulfiram suppresses pyroptosis by covalently modifying and inhibiting Gasdermin D (235). LDC7559 is a small molecule that was also proven to inhibit Gasdermin D and delay NETosis (236).

##### 5.1.4.5 Protein-kinase C inhibition

The protein-kinase C isoenzyme family plays a role in the downstream pathways of NETosis. The pan-PKC inhibition with Ro-31, PKC-α, and β-inhibitor Go 6976, and PKC-β inhibition with LY333531 reduce NET formation (237). PKC-δ inhibitor rottlerin prevented glucose-induces ERK activity and decreased TGF-β1-induced collagen synthesis in mesangial cells in an *in vitro* diabetic nephropathy model but it did not affect NET formation according to another study (237, 238).

**Table 2** summarizes the potential therapeutical targets of NET formation.

**Table 2:** Potential therapeutic targets of NETosis.

Disease-modifying antirheumatic drugs (DMARDs)		
Conventional synthetic DMARDs (csDMARDs)		
**Recombinant DNase**		dismantles NETs and accelerates clearance
**Toll-like Receptor inhibitors**	Hydroxychloroquine	inhibits NET formation by antagonizing Toll-like receptors (TLR-3, 7, 8, 9); has inhibitory effects on cyclic GMP-AMP synthase (cGAS), and has PAD4 inhibitory functions
	Chloroquine	inhibits NET formation by antagonizing Toll-like receptors (TLR-3, 7, 8, 9); reduces autophagy, and increases the pH in lysosomes, impeding antigen presentation on MHC class II molecules for CD4+ T-cells
	Enpatoran	dual TLR7/8 inhibitor
	GIT27 (VGX-1027)	targets primarily TLR4 but also interferes with TLR2, TLR6 signaling pathways in macrophages
	TAK-242	selective TLR4 inhibitor
	Eritoran	selective TLR4 inhibitor
**Calcineurin-inhibitors**	Cyclosporin	suppresses chemokinesis, adhesion, and angiotensin II response, hence reduces phagocytic activity, blocks T-cell overactivation and podocyte alteration
	Tacrolimus	suppresses chemokinesis, adhesion, and angiotensin II response, hence reduces phagocytic activity
	Voclosporin	suppresses chemokinesis, adhesion, and angiotensin II response, hence reduces phagocytic activity
**Colchicine**		inhibits immune response by dampening neutrophil chemotaxis, recruitment, adhesion, inflammasome activation, and NETosis.
**PAD-4 inhibitors**	Chlor-amidine	irreversibly blocks PAD4 active site via covalent modification
	GSK484	selectively and reversibly binds to PAD4
	GSK199	selectively and reversibly binds to PAD4
Targeted synthetic DMARDs (tsDMARDs)		
**JAK/STAT inhibitors**	Tofacitinib	decreases proteinuria, reduces mesangial cell proliferation and glomerular IgG deposition; in MRL/lpr mice reduces TGF-β receptor 1 in renal tissues, thus alleviates renal fibrosis
	Ruxolitinib	alleviates renal fibrosis in MRL/lpr mice model; lowers proinflammatory cytokine levels and regulates podocyte autophagy in diabetic kidney disease
	Baricitinib	decreases the urinary albumin/creatinine ratio from baseline at 6 months compared to placebo
	Peficitinib	
	Upadacitinib	
	Filgotinib	
	Brepocitinib	
**Tyrosine kinase inhibitors**	Bosutinib	Abl/Src inhibitor: reduces the FcγRIIa-mediated adhesion, neutrophil recruitment, and NET formation
	Dasatinib	reduces neutrophil and macrophage influx in skin wounds
**Bruton tyrosine kinase inhibitor**	Elsubrutinib	reduces proteinuria and improves glomerular histopathology in lupus-prone animal models
	Upadacitinib	reduces proteinuria and improves glomerular histopathology in lupus-prone animal models
**Spleen tyrosine kinase inhibitors**	Fostamatinib	highly selective Syk inhibitor, improves MPO-ANCA glomerulonephritis in a pre-clinical ANCA vasculitis model in Wistar Kyoto rats; delays renal progression in NZB/NZW mice in lupus nephritis model and anti-GBM glomerulonephritis in Sprague-Dawley and WKY rats
Biological DMARDs (bDMARDs)		
**Complement 5 inhibitors**	Eculizumab	inhibits complement-mediated thrombotic microangiopathy and significantly improves renal function in atypical HUS
	Avacopan	anti-C5a receptor antagonist
**Interferon pathway inhibitors**	Sifalimumab	monoclonal antibody: binds to interferon alpha and aids their neutralization
	Rontalizumab	monoclonal antibody: binds to interferon alpha and aids their neutralization
	Anifrolumab	human immunoglobulin: binds to the subunit 1 of interferon alpha receptor
**Depletion of B-lymphocytes**	Belimumab	anti-BlyS/BAFF, reduces circulating immunocomplexes
	Rituximab	anti-CD20, reduces circulating immunocomplexes
	Obinutuzumab	anti-CD20, reduces circulating immunocomplexes
**Interleukin antagonists**	Tocilizumab	anti-IL-6R antibody; improves endothelial function and decreases oxidative stress in RA, reduces the low-density granulocytes and NETosis
	Canakinumab	monoclonal antibody against IL-1β
	Anakinra	IL-1R blocker; reduces NET formation in a time- and dose-dependent manner, has a calcium-dependent role in the MPO and NE activation
	Rilonacept	soluble decoy receptor, which acts as an IL-1α and IL-1β trap
	GSK1070806	anti-IL-18 monoclonal antibody
	Rh-IL-18 BP	neutralizes IL-18 along with IL-37
**Other**	NI-0101	humanized monoclonal antibody against TLR4
Miscellaneous		
**Antioxidants**	N-acetylcysteine	modulates ROS production
	Amino salicylates	scavenges ROS superoxide, recovers SOD activity, NF-κB, MPO and proinflammatory cytokine production
	Metformin	mTOR and mtROS inhibitor; inhibits the NADPH oxidase and the membrane translocation of PKC-βII
	Idebenone	ubiquinone analog antioxidant; scavenges ROS, defends membranes from lipid peroxidation and restores ATP production in the mitochondria
	DPI	NADPH oxidase inhibitor; binds to the subunits and prevents electron flow and ROS production
	MitoTEMPO	Mitochondrial ROS scavenger; decreases spontaneous NETosis serum and anti-dsDNA levels, lowers proteinuria and renal IC deposition
**Vitamin D**		unknown pathomechanism
**Antibiotics**	Azithromycin	inhibits ROS
	Chloramphenicol	decreases MPO activity
	Gentamicin	unknown pathomechanism
**PKC inhibitors**	Ro-31-8220	pan-PKC inhibitor; blocks PMA-induced NET formation
	Go 6976	PKC-α and β inhibitor, reduces ROS production in PMA-induced NETosis
	LY333531	PKC inhibitor with a high selectivity for PKC-β, reduces ROS production by blocking the p47phox phosphorylation of NADPH-oxidase in PMA-induced NETosis
**PA-dPEG24**		peptide inhibitor of C1; dose-dependently blocks the MPO pathway of NET formation
**Prostaglandins**	PGE2	cAMP–PKA pathway modulators; limits NETosis in an exchange protein activated by cAMP- and protein kinase A-dependent manner (EP2 and EP4 Gαs-coupled receptors)
**Dibutyryl cAMP**		cell‐permeable cAMP analog
**Rolipram**		PDE4 inhibitor
**Butaprost**		EP2 receptor agonist
**LDC7599**		Gasdermin-D inhibitor; inhibits pore-formation in the nuclear and plasma membrane and prevents NETosis
**Disulfiram**		Gasdermin-D inhibitor; inhibits pore-formation in the nuclear and plasma membrane and prevents NETosis

AMP, adenosine monophosphate; ANCA, anti-neutrophil cytoplasmic antibodies; ATP, adenosine triphosphate; BAFF, B-cell activating factor; BLyS, B lymphocyte stimulator; cAMP, cyclic adenosine monophosphate; cGMP, cyclic guanosine monophosphate; dsDNA, double-stranded deoxyribonucleic acid; dsRNA, double-stranded ribonucleic acid; IC, immune complex; IL, interleukin; MHC, major histocompatibility complex; MPO, myeloperoxidase; mTOR, mammalian target of rapamycin; mtROS, mitochondrial reactive oxygen species; NADPH oxidase, nicotinamide adenine dinucleotide phosphate oxidase; NE, neutrophil elastase; NF-kB, nuclear factor kappa-light-chain-enhancer of activated B cells; PAD4, protein arginine deiminase 4; PDE4, phosphodiesterase-4 inhibitor; PKC-βII, protein kinase C beta II; PMA, phorbol myristate acetate; RA, rheumatoid arthritis; Rh-IL-18 BP, recombinant human IL-18 binding protein; ROS, reactive oxygen species; Syk, spleen tyrosine kinase; SOD, superoxide dismutase; TGF-β, transforming growth factor beta.

## 6 Conclusions

The pivotal role of neutrophil extracellular traps in the pathomechanism of renal disorders is an evolving hot topic (239, 240). NETs are not only generated by infectious stimuli, but they are also essential in several autoimmune and acute kidney disorders. A more in-depth understanding of the formation, regulation, and dysregulation of NETs offers potential therapeutic targets in kidney diseases leading to better patient and renal outcomes.

## Author contributions

MJ: Investigation, Visualization, Writing – original draft. AM: Investigation, Visualization, Writing – original draft. ZJ: Software, Writing – review & editing. NL: Conceptualization, Funding acquisition, Resources, Supervision, Writing – review & editing.
